# Controlled rate slow freezing with lyoprotective agent to retain the integrity of lipid nanovesicles during lyophilization

**DOI:** 10.1038/s41598-021-03841-4

**Published:** 2021-12-21

**Authors:** Eunhye Yang, Hyunjong Yu, SungHak Choi, Kyung-Min Park, Ho-Sup Jung, Pahn-Shick Chang

**Affiliations:** 1 Department of Agricultural Biotechnology, Seoul National University, Seoul, 08826 Republic of Korea; 2 Research Institute of Agriculture and Life Sciences, Seoul National University, Seoul, 08826 Republic of Korea; 3 Center for Agricultural Microorganism and Enzyme, Seoul National University, Seoul, 08826 Republic of Korea; 4 Center for Food and Bioconvergence, Seoul National University, Seoul, 08826 Republic of Korea; 5 Department of Food Science and Biotechnology, Wonkwang University, Iksan, 54538 Republic of Korea

**Keywords:** Design, synthesis and processing, Colloids, Biological techniques, Nanoparticles

## Abstract

We designed a novel lyophilization method using controlled rate slow freezing (CSF) with lyoprotective agent (LPA) to achieve intact lipid nanovesicles after lyophilization. During the freezing step, LPA prevented water supercooling, and the freezing rate was controlled by CSF. Regulating the freezing rate by various liquid media was a crucial determinant of membrane disruption, and isopropanol (freezing rate of 0.933 °C/min) was the optimal medium for the CSF system. Lyophilized lipid nanovesicle using both CSF and LPA retained 92.9% of the core material and had uniform size distributions (Z-average diameter = 133.4 nm, polydispersity index = 0.144), similar to intact vesicles (120.7 nm and 0.159, respectively), after rehydration. Only lyophilized lipid nanovesicle using both CSF and LPA showed no changes in membrane fluidity and polarity. This lyophilization method can be applied to improve storage stability of lipid nanocarriers encapsulating drugs while retaining their original activity.

## Introduction

Lipid nanovesicles, which are spherical particles formed from lipid bilayers enclosing discrete aqueous spaces, serve as nanocarriers of biologically and therapeutically active compounds for biodistribution to target sites^1,2^. Lipid nanovesicles can improve the pharmacokinetics and biodistribution of theragnostic compounds because of their inherent advantages, including high compound-loading efficiency, stability in biological environments, controllable release kinetics, and biocompatibility^3,4^. Hence, lipid nanovesicles have been used commercially as drug delivery systems, carriers for medical diagnostics^5,6^, mRNA vaccine delivery systems with minimal toxicity^7,8^, signal enhancers in analytical biochemistry^9^, and solubilizers for antibiotics to kill pathogens^10–15^.

Lipid nanovesicles contain large amounts of unsaturated fatty acids, which can cause instability in the membrane structure depending on the external conditions, limiting their commercial application. Modification of the membrane surface^11,12^, grafting polymers to the lipid head group^13^, and solidification via lyophilization and spray drying have been employed to improve the physicochemical stability of lipid nanovesicles. Lyophilization is widely used for the solidification and storage of vesicle formulations as it prevents degradation, aggregation, or fusion of the nanovesicles by reducing interactions between the lipid membrane and water, thus improving stability^14–16^. Although lyophilization has several advantages over other solidification methods, lipid bilayer membranes can be damaged during lyophilization because of the physical stress caused by the formation of ice crystals during the freezing step^16^. Attempts to improve the integrity of lipid bilayer membranes by incorporating a lyoprotective agent (LPA) in the lipid nanovesicle solution to support a frozen matrix have been unsuccessful in terms of maintaining intact nanovesicle size distributions and morphology. These results suggest that the use of LPA alone is insufficient to retain the integrity of membranes during lyophilization.

The freezing rate considerably affects membrane structure during lyophilization, which causes differences in ice crystal growth. Quick freezing (e.g., freezing rate of 70 °C/min), in which small-volume suspensions are immersed in liquid nitrogen, reduces physical stress and metastable behavior via the formation of fine ice crystals^17^. However, extremely quick freezing creates an osmotic shock because of the difference in freezing point between internal and external aqueous phases in lipid nanovesicles^18^. Meanwhile, slow freezing (e.g., freezing rate of 0.5 °C/min) reduces the osmotic pressure of the system, and water molecules can diffuse slowly across lipid bilayers^19,20^. However, the ice crystal size in lipid vesicles is larger with slow freezing compared with quick freezing; larger ice crystals cause physical damage to the lipid bilayer due to volume expansion of the ice^21^. Hence, precise control of the freezing rate is necessary to diminish disruption of lipid bilayer membranes by ice crystal formation.

Herein, we designed a novel lyophilization method using LPA and a controlled rate slow freezing (CSF) system to adjust the ice crystal growth of lipid nanovesicle solutions. Lipid nanovesicles were prepared from a soy-phosphatidylcholine lipid based on the microfluidic principle. Trehalose and sucrose were used as the LPA for the internal and external aqueous phases of the lipid nanovesicles, respectively. In the CSF system, organic solvents with a freezing point lower than − 75 °C (deep freezing condition) were used as liquid media.

## Materials and methods

### Materials

Soy phosphatidylcholine (soy-PC; lecithin from soybean) was purchased from Ilshin wells (Seoul, Korea). Cholesterol (CH; > 99% purity), trehalose dihydrate, sucrose, and calcein were purchased from Sigma-Aldrich Co. (St. Louis, MO, USA). 1,6-Diphenyl-1,3,5-hexatriene (DPH) and 6-dodecanoyl-*N*,*N*-dimethyl-2-naphthylamine (Laurdan) were acquired from Molecular Probes (Eugene, OR, USA). Acetone, methanol, 1-propanol, and isopropanol (IPA) were purchased from Daejung (Siheung, Gyeonggi-do, Korea). All other chemicals were obtained from Sigma-Aldrich Co. and used without further purification.

### Preparation of lipid nanovesicles with lyoprotective agent

Primary water-in-oil (W_1_/O) emulsions were prepared by emulsification. The W_1_ phase was prepared by dissolving trehalose (15 mM), a lyoprotective agent (LPA) for the internal aqueous phase of lipid nanovesicles, in distilled water. Calcein (0.4 mM), a fluorescent probe typically used to be encapsulated within the internal aqueous phase of lipid nanovesicles, was added to the W_1_ phase. The oil phase comprised a mixture of soy-PC and CH to a molar ratio of 4:1 with a total lipid content of 3 mmol; soy-PC and CH were dissolved in ethyl acetate/*n*-hexane (4:1, v/v) and stirred for 5 min to form a solution containing the lipids. To form coarse inverted micelles, the mixture was homogenized for 5 min via sonication using a probe-type ultrasonicator (ULH-700S, Jeiotech, Korea). Then, to produce nano-size inverted micelles, the coarse micelle solution was homogenized in 10 cycles at 100 MPa using a high-pressure homogenizer (MN400BF, Micronox, Seongnam, Korea) primed with organic solvents. The nano-size inverted micelles were mixed with 600 mL of distilled water and 15 mmol sucrose, which was used as the LPA for the external aqueous phase of the lipid nanovesicles. To prepare the nano-size double emulsion of water-in-oil-in-water (W_1_/O/W_2_), the coarse double emulsion solution was homogenized in five cycles at 30 MPa using the high-pressure homogenizer primed with distilled water. After homogenization, the W_1_/O/W_2_ emulsion was stirred at room temperature for 24 h to evaporate organic solvents between the lipid bilayers, producing lipid nanovesicles.

### Lyophilization of lipid nanovesicles

For controlled rate slow freezing (CSF) system, a vessel containing liquid media was placed in a deep freezer at − 75 °C for 4 h, enabling the medium temperature to reach equilibrium at − 75 °C. Then, 30 mL of lipid nanovesicle solution in a 50 mL polystyrene tube was immersed in the pre-cooled liquid medium and frozen at − 75 °C for 8 h. Conventional freezing was performed by placing the lipid nanovesicle solution directly into the deep freezer for 8 h. After freezing step, the freeze-drying process was performed only the primary drying using a freeze dryer (FD8512, Ilshin Lab Co., Ltd., Seoul, Korea) at − 75 °C for 48 h. The chamber pressure was maintained at 20 Pa during the freeze-drying process. The tubes containing the lyophilized lipid nanovesicles were immediately sealed and stored at 4 °C until use.

### Characterization of lipid nanovesicles

To characterize the lyophilized lipid nanovesicles after reconstitution, 100 mg of the lyophilized lipid nanovesicles, prepared by 11.9 mL of lipid nanovesicle solution, were rehydrated by adding 10 mL of distilled water and shaking gently using vortex mixer for 1 min. Following rehydration, the mean diameters of the lipid nanovesicles were measured using a dynamic light scattering instrument (Zetasizer Nano ZS, Malvern, UK) with a submicron particle size analyzer. The sample measurement conditions were as follows: refractive index 1.330, viscosity 0.8872 cP, equilibration time 1 min, measurement temperature 25 °C, and measurement angle 173° backscattering.

For transmission electron microscopy (TEM), the lipid nanovesicles were stained with uranyl acetate as a negative staining reagent. Then, 10 µL of lipid nanovesicle sample was dropped onto a formvar-coated carbon grid (200 mesh). After 1 min, the grid was loaded with 2% uranyl acetate solution (w/v) and incubated for 1 min, and then washed with double-distilled water. The grid was dried at room temperature before the lipid nanovesicles were visualized by TEM (80 keV, JEOL Ltd., Tokyo, Japan).

### Evaluation of membrane fluidity and polarity

The inner membrane fluidity of the lipid nanovesicles was evaluated as described previously^22,23^. The fluorescent probe DPH was added to the lipid nanovesicle suspension at a molar ratio of 250:1, lipid:DPH; the final concentrations of lipid and DPH were 100 and 0.4 μM, respectively. The solution was incubated at 30 °C for 30 min, then the fluorescence polarization of DPH (*excitation* = 360 nm, *emission* = 430 nm) was measured using a fluorescence spectrophotometer (FP-8500, JASCO, Tokyo, Japan). The sample was excited with vertically polarized light (360 nm), and the emission intensities perpendicular (*I*_⊥_) (0°, 0°) and parallel (*I*_||_) (0°, 90°) to the excited light were measured at 430 nm. The polarization (P) of DPH was calculated using the following equations:

$$\begin{aligned} P &= ( {I_{\parallel } - GI_{ \bot } } )/( {I_{\parallel } + GI_{ \bot } } ), \\ G& = i_{ \bot } /i_{\parallel }, \end{aligned}$$

where *I*_‖_ and *I*_⊥_ are the emission intensities parallel and perpendicular to the horizontally polarized light, respectively. *G* is the correction factor, and *i*_⊥_ and *i*_‖_ are the emission intensities perpendicular and parallel to the vertically polarized light, respectively. The membrane fluidity was evaluated based on the reciprocal of polarization, *1/P*.

The fluorescent probe Laurdan is sensitive to polarity, enabling the surface polarity of lipid membranes to be determined^24–26^. Laurdan emission spectra exhibit a red shift caused by dielectric relaxation. Thus, the emission spectra were calculated by obtaining the general polarization (*GP*_340_) for each emission wavelength as follows:

$$GP_{340} = ( I_{440} - I_{490} )/(I_{440} + I_{490} ),$$

where *I*_440_ and *I*_490_ represent the emission intensities of Laurdan excited with 340 nm light. The total concentrations of lipid and Laurdan in the test solution were 200 µM and 1 μM, respectively.

### Determination of encapsulation efficiency

The encapsulation efficiency and core material retention of the lipid nanovesicles were determined fluorometrically using a previously developed method^27^. Briefly, 25 μL of resuspended lipid nanovesicles (10 mg lipid/mL) were diluted to 500 μL with 50 mM MOPS buffer (pH 7.0) containing 110 mM sodium chloride. Cobalt chloride (CoCl_2_) was then added to quench the fluorescence of external calcein to eliminate background noise. Then, the lipid nanovesicles were lysed with detergent to determine the background fluorescence at zero encapsulated volume. Fluorescence intensity was measured before (*F*_b_) and after (*F*_a_) the addition of 5 μL of 10 mM CoCl_2_, and before and after the addition of 25 μL 10% Triton X-100 (*F*_totq_), at 490 nm excitation and 520 nm emission. Encapsulation efficiency was calculated as follows:

$${\text{Encapsulation efficiency}} \, ({\%}) = ( {F_{a} - F_{totq} })/({F_{b} - F_{totq} }) \times 100.$$

### Temperature profiling

The temperature profile was measured using a multi-channel thermometer (Dr.meter, Elitech, Taiwan). The temperature was measured every 1 min. The temperature profiles of the lipid nanovesicle solutions during conventional freezing and CSF were measured by placing the thermometer sensor inside the sample at the center. The degree of supercooling was defined as the difference between the temperature at which ice crystals first form and the equilibrium freezing point of the solution^28,29^. The duration of ice crystal growth was defined as the length of time at which the temperature of the solution fell within the zone of ice crystal formation (− 5 to 0 °C)^30^.

## Results and discussion

### Freezing behavior of lipid nanovesicles

**Figure 1:**
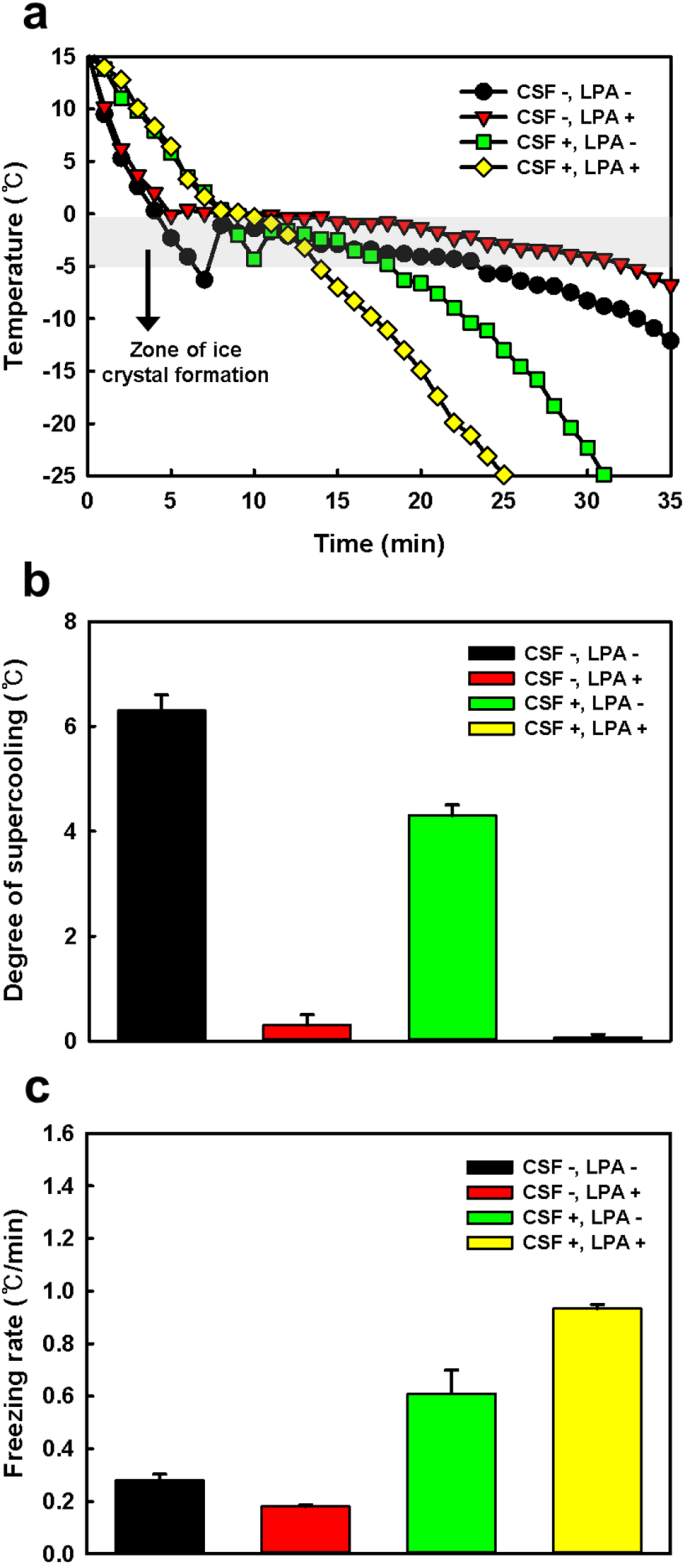
Effects of CSF and LPA on the freezing behavior of the lipid nanovesicle solution. (**a**) Temperature profiles, (**b**) degree of supercooling, and (**c**) freezing rate under different freezing conditions. Isopropanol was used as the liquid medium for the CSF system. The freezing rate was measured within the zone of ice crystal formation (− 5 to 0 °C).

As shown in Fig. 1a, the effects of CSF and LPA on ice crystal formation were assessed by plotting thermocouple temperature profiles of the lipid nanovesicle solution within the zone of ice crystal formation (− 5 to 0 °C). The degree of supercooling and the freezing rate, which determine ice crystal formation, were measured in the lipid nanovesicle solution (Supplementary Fig. S1). The freezing process firstly involves ice nucleation, in which a minuscule crystalline lattice structure is formed. Once stable ice nuclei are formed, ice crystal growth proceeds by the addition of molecules to the interface^31^. Ice nucleation is affected by the supercooling of water, which is the retention of the liquid state below its equilibrium freezing point (0 °C). The freezing rate, the velocity to pass through the critical zone of ice crystal formation (− 5 to 0 °C), determines the ice crystal size and distribution.

The degree of supercooling of the lipid nanovesicle solution was decreased by approximately half in the CSF system compared with the conventional freezing system (Fig. 1b). Meanwhile, the degree of supercooling of the solution was significantly lower with the addition of LPA than without LPA, indicating that supercooling was controlled primarily by LPA treatment. The presence of solutes such as LPA generally causes a decrease in the freezing point of solutions and promotes heterogeneous nucleation, which lead to a decrease in the degree of supercooling^32,33^. The combination of CSF and LPA completely suppressed supercooling of the lipid nanovesicle solution. The lowered degree of supercooling suggested that faster ice nucleation occurred in the solution, which contributed to a decrease in ice crystal size^34,35^.

The freezing rate of the lipid nanovesicle solution in the conventional freezing system was 0.279 °C/min, and it increased after adding LPA to the solution, which indicated that LPA treatment had little impact on ice crystal growth (Fig. 1c). On the other hand, the freezing rates of the lipid nanovesicle solution in the CSF system with and without LPA were 0.933 and 0.610 °C/min, respectively. Compared with the conventional freezing system, the CSF system increased the freezing rate with and without LPA by 5.2- and 2.2-fold, respectively.

Generally, freezing at high rates reduces the time for ice crystals to grow in solution, thereby forming small and uniform ice crystals. Extremely quick freezing using liquid nitrogen can decrease the size of ice crystals more dramatically compared with freezing using the CSF system, but quick freezing also disrupts lipid bilayer membranes by creating high osmotic pressure^36^. Hence, freezing at a moderate rate (i.e., not too quick or too slow freezing) forms fine ice crystal and concurrently leads the appropriate diffusion from the aqueous phase of lipid nanovesicle^37,38^. These suggest that the CSF system regulating the freezing rate can minimize the membrane disruption from ice crystal formation.

### Effects of freezing rate on lipid nanovesicles

**Figure 2:**
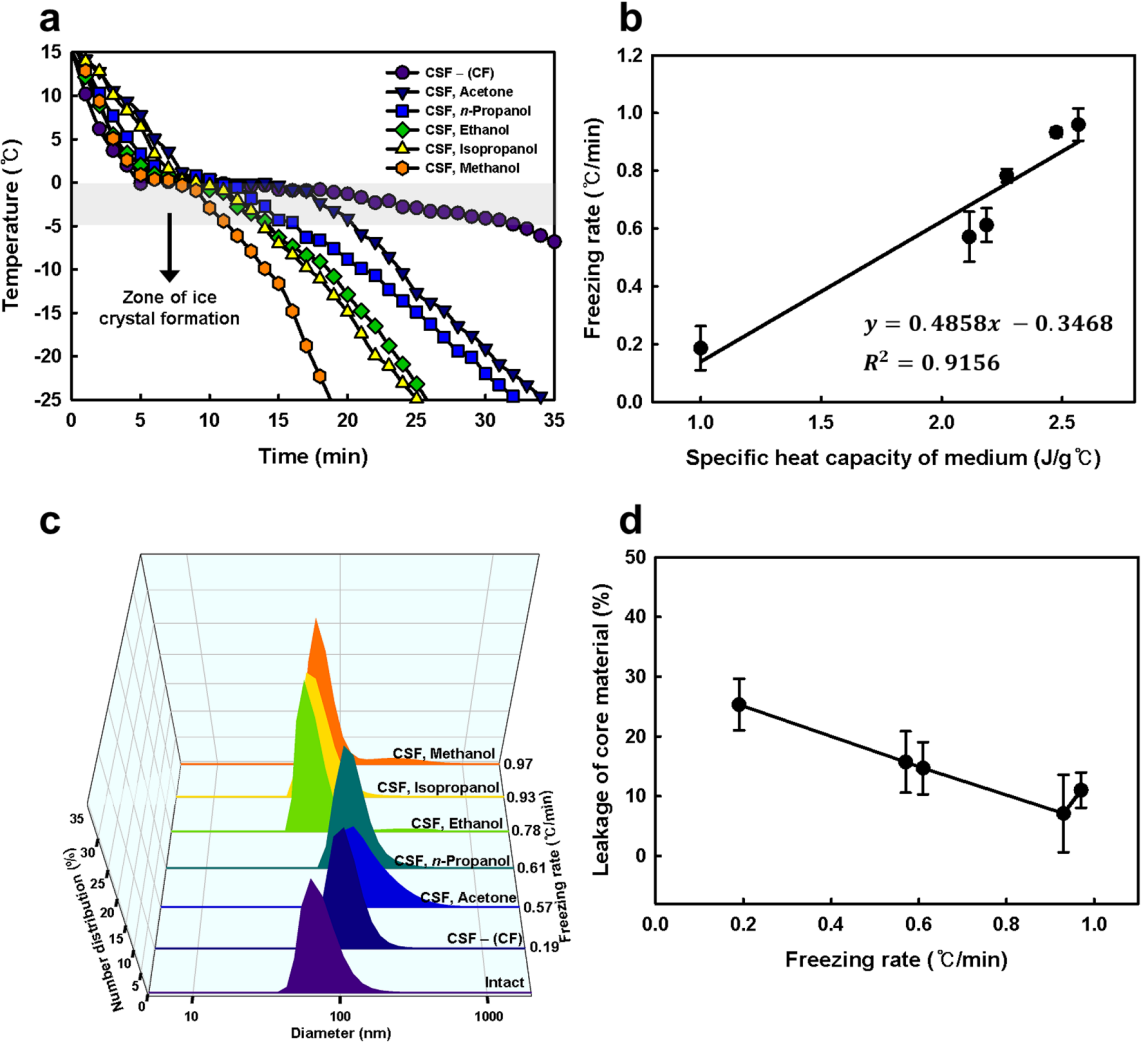
Effects of the medium (organic solvent) in the CSF system on the freezing rate and physicochemical properties of lipid nanovesicles. (**a**) Temperature profiles of lipid nanovesicles in the conventional freezing and CSF systems with different media. (**b**) Correlation between the specific heat capacity of the medium and freezing rate of lipid nanovesicles. (**c**) Size distribution and (**d**) leakage of encapsulated material (fluorescent calcein) of lyophilized lipid nanovesicles in the various freezing systems with different freezing rates. The size distribution and leakage of lyophilized lipid nanovesicles were measured after rehydration.

Various organic solvents (e.g., acetone, *n*-propanol, ethanol, isopropanol, and methanol) as the liquid media were applied to the CSF system to control the freezing rate in detail. The temperature profiles of the lipid nanovesicle solutions in the CSF system using organic solvents showed that the freezing rate differed substantially depending on the liquid medium (Fig. 2a). The lowest freezing rate (0.186 °C/min) was obtained using the conventional freezing system, whereas the highest freezing rate (0.966 °C/min) was obtained using the CSF system with methanol as the liquid medium.

The freezing rate in the CSF system was significantly correlated with the specific heat capacity of the medium (Table S1). Linear regression indicated that the freezing rate increased proportionally with the specific heat capacity of the medium (Fig. 2b). Changes in the freezing rate were significantly associated with the removal of latent heat of fusion produced by the phase change of water from a liquid to crystalline form. These results suggest that as the specific heat capacity of the liquid medium increases, a higher driving force for heat transfer is provided to the sample solution, which increases the freezing rate accordingly. Furthermore, it is possible to precisely control the freezing rate by simply changing the liquid medium in the CSF system (Supplementary Fig. S2).

The effect of the freezing rate on the integrity of the lipid bilayer membrane was evaluated by measuring the size distribution of lipid nanovesicle after lyophilization (Fig. 2c). The intact lipid nanovesicle prior to lyophilization had a Z-average diameter (D_z_) of 120.7 nm and polydispersity index (PDI) of 0.159, and transmission electron microscopy (TEM) images showed a monodispersed morphology of the lipid nanovesicles (Supplementary Fig. S3). Compared with the size distribution of intact lipid nanovesicles, changes in D_z_ and PDI were observed in all of the lyophilized lipid nanovesicles. However, the changes in the size distributions of the lyophilized lipid nanovesicles differed significantly among the different freezing systems. The D_z_ and PDI of lyophilized lipid nanovesicles showed the greatest changes in the conventional freezing and CSF systems with acetone and the smallest changes in the CSF system with isopropanol, suggesting that the change in size distribution decreased as the freezing rate increased (Supplementary Fig. S4). Nevertheless, lyophilized lipid nanovesicles in the CSF system with methanol, in which the freezing rate was highest, showed a greater increase in D_z_ compared with the CSF system with isopropanol.

These results suggest that too quick freezing leads to disruption of the lipid bilayer membrane, and the membrane integrity of lipid nanovesicles can be maintained completely after lyophilization when the appropriate freezing rate is applied. It is well documented that too quick freezing, such as immersion of small volume suspension in liquid nitrogen, reduces disruption of the lipid bilayer membrane, since the freezing results in the formation of fine ice crystal and homogenous dispersion of LPA. However, several studies have shown that the lipid nanovesicles freeze-dried after quick freezing using liquid nitrogen have lower retention of core material than slow freezing^37,39^. Ice crystal formation in the inner aqueous phase of lipid nanovesicle can be minimized by slow freezing which provides more time to recover from deformations produced by osmotic pressure. Moreover, slow freezing reduces the amount of water in the glass matrix due to the freeze-concentration and, thus, a higher glass transition temperature is obtained^38^. However, the effect of freezing rate on the membrane integrity is strongly dependent on the lipid composition and the presence of cholesterol.

We assessed leakage of encapsulated material (fluorescent calcein in the internal aqueous phase) in lipid nanovesicles after lyophilization at different freezing rates (Fig. 2d). The highest leakage (25.32%) was measured in lyophilized lipid nanovesicles in the conventional freezing system. Leakage from lyophilized lipid nanovesicles decreased as the freezing rate increased. But, the leakage in the CSF system using isopropanol showed the least leakage (7.11%), consistent with the freezing rate-dependent changes in size distribution. These results demonstrate that minute changes in the freezing rate considerably affect the integrity of lipid bilayer membranes during freezing; thus, precise control of the freezing rate is crucial for maintaining the lipid nanovesicle structure. Isopropanol was selected as the optimal liquid medium in the CSF system for lyophilization of the lipid nanovesicles.

### Powderization and rehydration of lipid nanovesicles

Powderization of lipid nanovesicles via lyophilization is a promising technology that can improve long-term stability and transportability for practical applications^38,40^. Lyophilized lipid nanovesicles must be reconstituted by adding a solution for rehydration prior to administration. The rehydration behavior of the lipid nanovesicle powder is important for product quality in terms of practical applications^41^. Hence, we examined the effects of CSF and LPA on the powderization and rehydration behavior of lipid nanovesicle powders.

**Figure 3:**
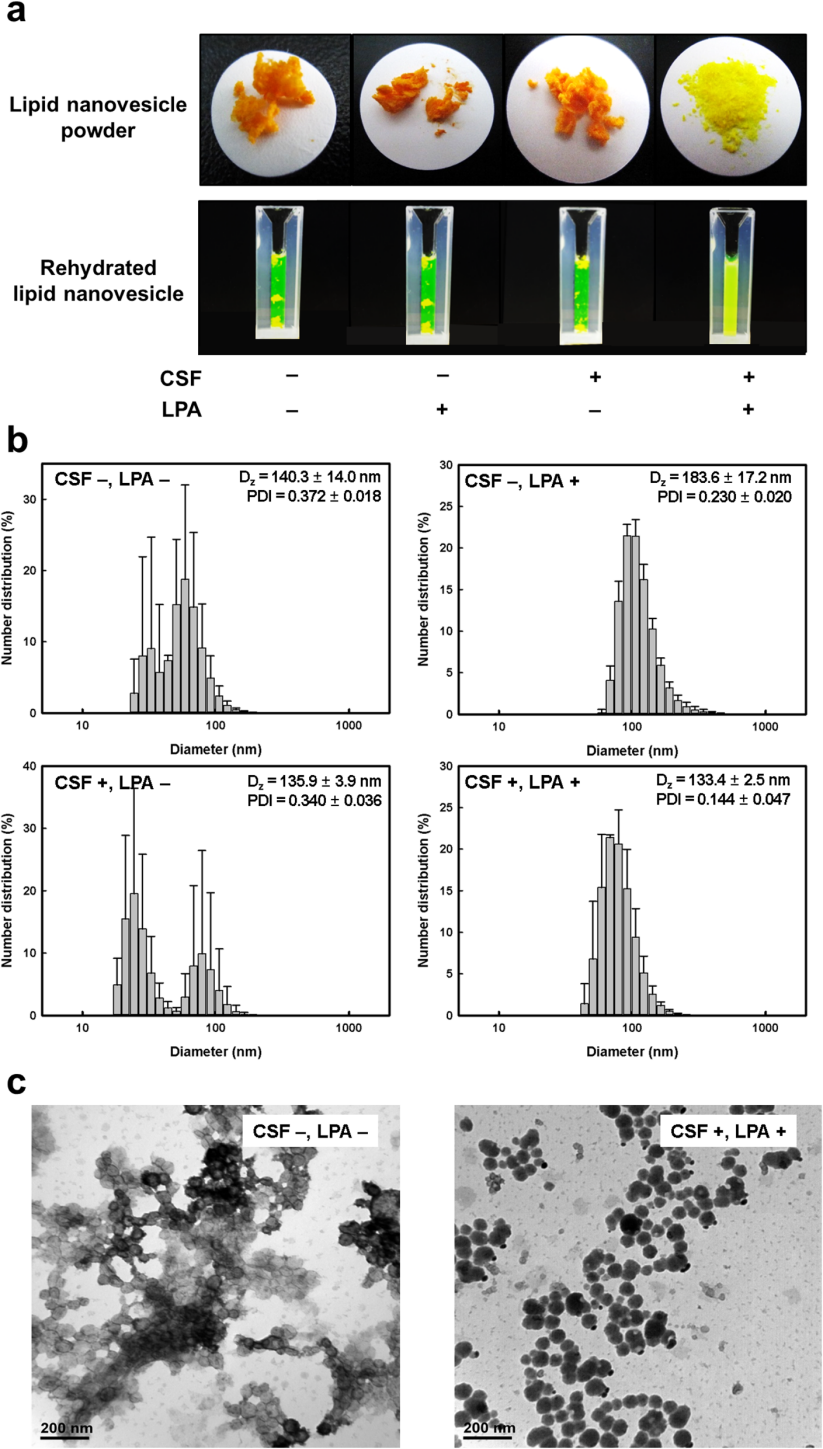
Effects of CSF and LPA on the rehydration behavior of lipid nanovesicle powders produced by lyophilization. (**a**) Appearance of lipid nanovesicle powders and rehydrated lipid nanovesicles. (**b**) Size distribution and (**c**) TEM images of lipid nanovesicle powders after rehydration.

As shown in Fig. 3a, the lipid nanovesicle powder lyophilized in the conventional freezing system formed a sticky agglomeration that did not completely disperse upon rehydration, regardless of LPA treatment. Lyophilization in the CSF system without LPA produced aggregated powder, which had low dispersibility following rehydration. By contrast, the powder produced by lyophilization in the CSF system with LPA had a fine and flour-like consistency and was dispersed completely in the solution without aggregation.

LPA such as sucrose and trehalose protects lipid nanovesicles during freezing process (i.e., cryoprotection), which influences the ice crystal formation. However, during the drying process, the disaccharides usually prevents the damages on the lipid bilayer membrane and, therefore, encourages rehydration of lipid nanovesicles by maintaining the lipid in the liquid crystalline phase at room temperature, even when dry. It has been proposed that the sugars depress the phase transition in dry phospholipids by hydrogen bonding to the polar headgroup, known as the water replacement hypothesis^42^.

The size distributions of the lipid nanovesicle powders differed from those of intact lipid nanovesicles after rehydration, except for the powder produced by lyophilization using CSF with LPA (Fig. 3b and Supplementary Fig. S5). The lipid nanovesicle powder without LPA showed two size distribution peaks after rehydration, regardless of the freezing system. These results indicated that LPA prevented the collapse, fusion, or aggregation of lipid nanovesicles during lyophilization, during which the water molecules that interacted with the polar phosphate groups of lipid bilayers were displaced by LPAs such as saccharides. The surface of the frozen vesicles was covered by a concentrated aqueous saccharide solution or glassy solid, which prevented physical damage by ice crystals causing disruption of the lipid bilayer^43^. Meanwhile, the lipid nanovesicles lyophilized by conventional freezing (without CSF) with LPA showed 1.52- and 1.45-fold increases in D_z_ and PDI after rehydration, respectively, compared with the intact lipid nanovesicles. Monodispersed size distributions of lipid nanovesicles similar to those of the intact vesicles were obtained only after lyophilization by CSF with LPA after rehydration. These results suggest that both the LPA treatment and CSF system are required to retain the integrity of lipid bilayer membranes during lyophilization and rehydration.

TEM images of the lipid nanovesicles lyophilized by conventional freezing without LPA and those lyophilized by CSF with LPA after rehydration are shown in Fig. 3c. Aggregations and structural disruptions were observed in the lipid nanovesicles lyophilized by conventional freezing in the absence of LPA. By contrast, the lipid nanovesicles lyophilized by CSF with LPA reverted to a spherical structure after rehydration, similar to intact lipid nanovesicles.

### Membrane fluidity and polarity of rehydrated lipid nanovesicles

**Figure 4:**
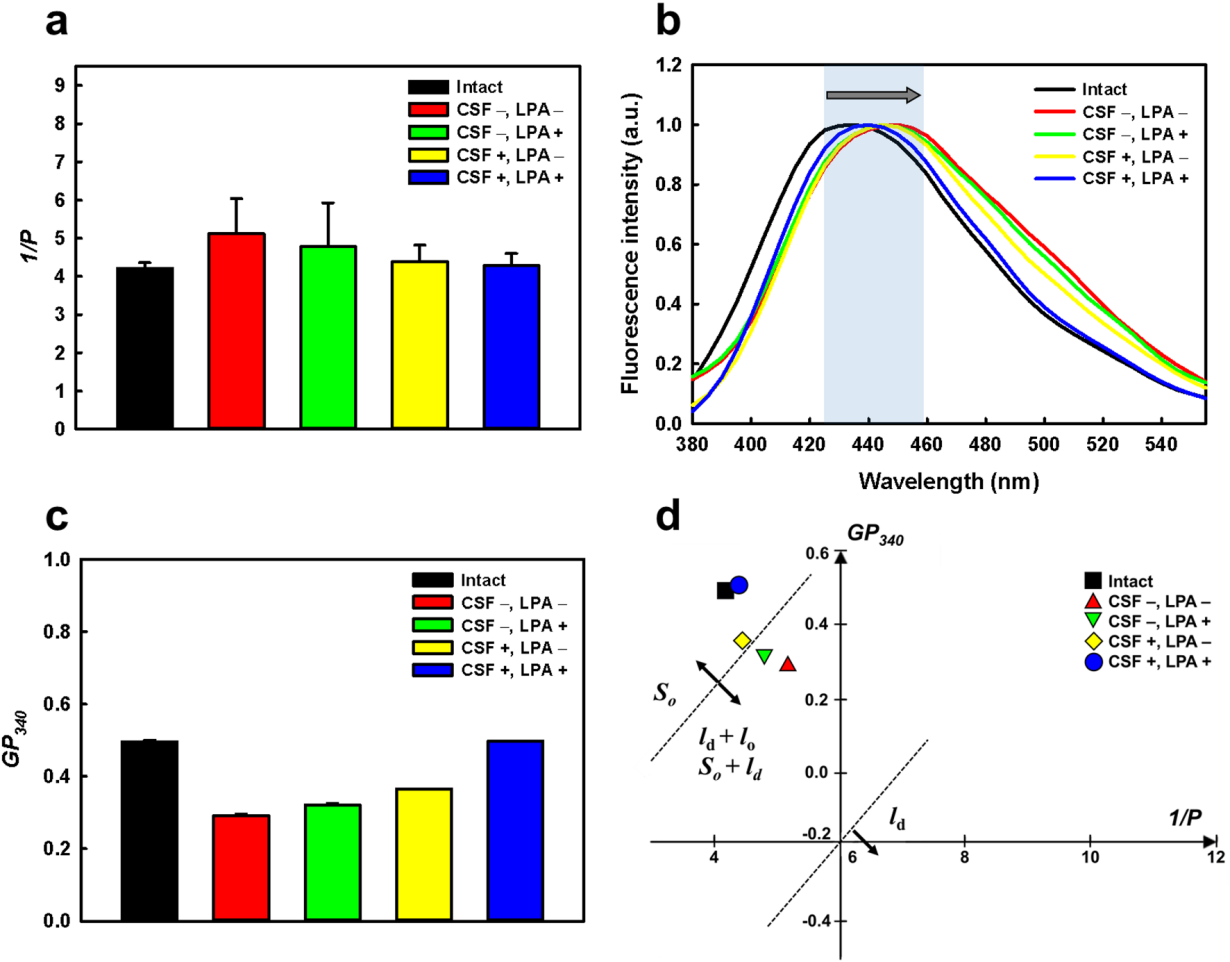
Membrane fluidity and polarity of intact lipid nanovesicles and lyophilized lipid nanovesicles with different freezing conditions, after rehydration. (**a**) The membrane fluidity (*1/P*) values of the lipid nanovesicles measured using 1,6-diphenyl-1,3,5-hexatriene fluorescent dye. (**b**) Fluorescence spectra of Laurdan intensity and (**c**) the membrane polarity (*GP*_340_) values of the lipid nanovesicles. Total concentrations of lipid and Laurdan in the test solution were 200 and 1 μM, respectively. (**d**) Cartesian diagram of the lipid nanovesicles. The *x* and *y* axes indicate *1/P* and *GP*_340_ values, respectively.

The effects of CSF and LPA on membrane integrity of lyophilized lipid nanovesicles were assessed by measuring membrane fluidity and polarity after rehydration. The membrane fluidity (*1/P*) values of rehydrated lipid nanovesicles after lyophilization with different freezing conditions (with or without CSF and LPA) were measured, and the results indicated membrane fluidity (Fig. 4a). The *1/P* values of lipid nanovesicles lyophilized in the CSF system, regardless of LPA treatment, were similar to those of the intact nanovesicles, suggesting that lyophilization in the CSF system did not cause considerable changes in the membrane fluidity of lipid nanovesicles. However, the *1/P* values of lipid nanovesicles lyophilized in the conventional freezing system (without CSF) in the presence or absence of LPA were higher than those of intact nanovesicles.

Membrane polarity of lyophilized lipid nanovesicles with different freezing conditions was determined using Laurdan, a fluorescence molecular probe. Laurdan shows specific emission peaks at 440 and 490 nm that originate from lipid bilayer membranes in ordered (*l*_o_, *S*_o_) and disordered (*l*_d_) phases, respectively. Figure 4b shows the fluorescence spectra of Laurdan in lipid nanovesicles lyophilized under different freezing conditions. An emission peak shift of Laurdan toward longer wavelengths (red shift) occurred in all lyophilized lipid nanovesicles, compared with the spectrum from intact lipid vesicles, but the peak shifts differed depending on the freezing conditions. Lyophilized lipid nanovesicles showed the lowest shift in the CSF system with LPA and the highest shift in the conventional freezing system without LPA. These results indicated that the freezing conditions determined the changes in the phase state of lipid membranes after lyophilization.

The membrane polarity (*GP*_340_) values of the lyophilized lipid nanovesicles were measured to identify the degree of hydration of the membrane surface (Fig. 4c). The *GP*_340_ value of the intact lipid nanovesicles was approximately 0.5, whereas that of lyophilized lipid nanovesicles without LPA or CSF decreased significantly after rehydration. The decrease in the *GP*_340_ value of lipid vesicles implied that the *l*_d_ phase increased, and the ordered (*S*_o_ or *l*_o_) phase decreased, in the lipid membrane. Only lyophilized lipid nanovesicles treated with both LPA and CSF showed no change in the *GP*_340_ value compared with intact vesicles. These results suggest that no fusion occurs, and the lipids remain in *l*_o_ phase when lipid nanovesicles are lyophilized with both LPA and CSF. In other words, lyophilization with both LPA and CSF allows lipid nanovesicles to retain the trapped solute during rehydration since the vesicles do not undergo a phase transition.

In Fig. 4d, the membrane fluidity and polarity of lyophilized lipid nanovesicles are plotted as a Cartesian plot, in which the phase state of the lipid membranes can be classified based on the *GP*_340_ and *1/P* values. The cross point of the *x* and *y* axes is the threshold point of the phase transition in the soy-phosphatidylcholine lipid vesicle (*1/P* = 6.0, *GP*_340_ = − 0.2)^44,45^. Using Cartesian diagram analysis, lipid vesicles with lower fluidity (*1/P* < 6) and higher polarity (*GP*_340_ > − 0.2) were plotted in the second quadrant, indicating that lipid membranes are in the *S*_o_ or *l*_o_ phase. The intact and lyophilized lipid nanovesicles in the CSF system existed in a solid-ordered (*S*_o_) phase with a lower fluidity and higher *GP*_340_ value compared with lyophilized lipid nanovesicles in the conventional freezing system. Lyophilized lipid nanovesicles in the conventional freezing system, regardless of LPA treatment, were found near the boundary of the *S*_o_/*l*_o_ and *l*_d_ phases, indicating that they are in *l*_d_ phase partially mixed with the *S*_o_ or *l*_o_ phase. The plot showed that lipid membranes of intact and lyophilized lipid nanovesicles in the CSF system existed in the *S*_o_ phase, with a lower fluidity and higher *GP*_340_ value than those of the other nanovesicles. Meanwhile, the lyophilized lipid nanovesicles in the conventional freezing system were found near the boundary of the *S*_o_ and *l*_d_ phases, indicating that their lipid membranes are in a *l*_d_ phase partially mixed with the *S*_o_ or *l*_o_ phase.

**Figure 5:**
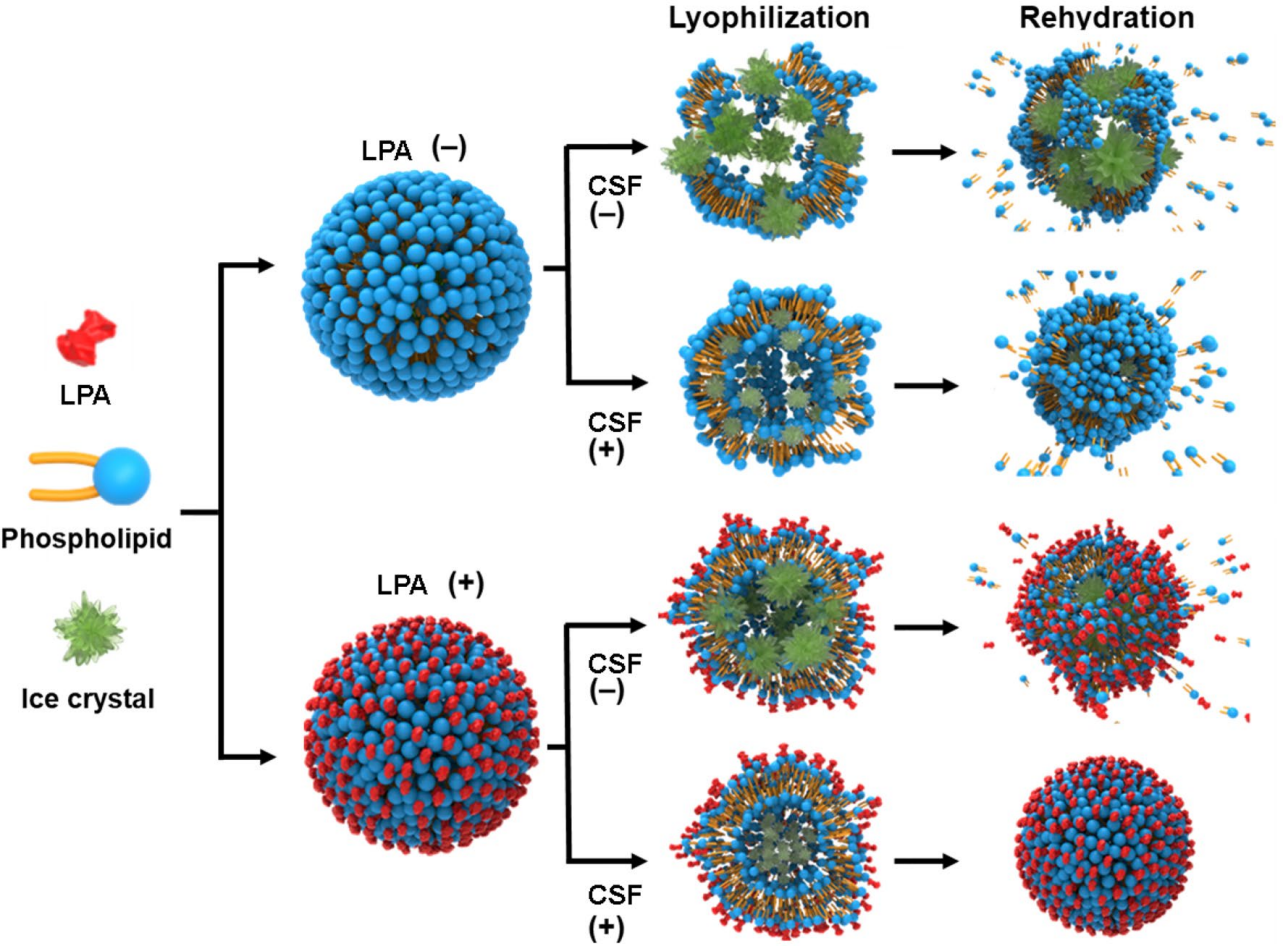
Schematic illustration representing the effects of LPA and CSF on lipid bilayer membranes during lyophilization and rehydration.

These results suggest that the phase state of the lipid membrane was significantly altered during lyophilization in the conventional freezing system, which might be attributed to the formation of large and heterogeneous ice crystals, inducing membrane fusion of lipid nanovesicles. Nevertheless, only the lipid nanovesicles lyophilized with both LPA and CSF were found to be similar to intact nanovesicles in terms of the phase state of the membrane. These results suggest that membrane integrity is retained during lyophilization and completely reconstituted by rehydration only when both CSF and LPA are applied during the freezing step. The effects of LPA and CSF on the integrity of lipid nanovesicles during lyophilization and rehydration are presented in Fig. 5.

### Storage stability of lyophilized powder

**Figure 6:**
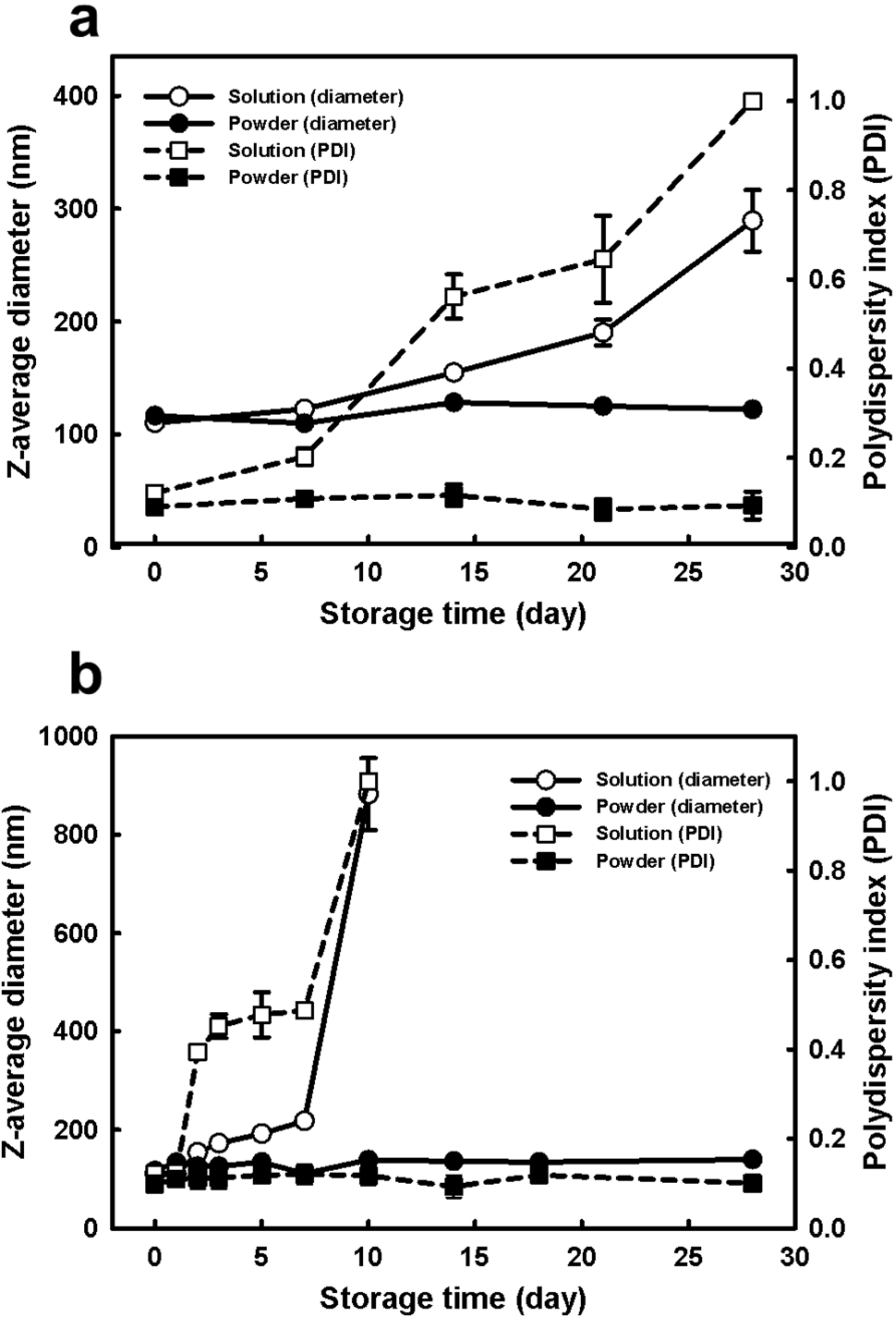
Storage stability of lipid nanovesicle solutions and lyophilized lipid nanovesicle powders incubated at (**a**) 4 °C and (**b**) 30 °C. The lyophilized sample was produced via lyophilization with lyoprotective agent (LPA) and controlled rate slow freezing (CSF) using isopropanol. Diameter, Z-average diameter; PDI, polydispersity index.

Lyophilization using LPA and CSF achieved optimal recovery of the lipid nanovesicle powder after long-term storage (Fig. 6). During storage at 4 °C and 30 °C for 30 days, the lipid nanovesicle powder retained its monodispersed distribution and colloidal stability unlike vesicles stored in solution (without lyophilization), which became unstable and aggregated after a short period (2 or 7 days). These results confirmed that this lyophilization method preserves intact lipid bilayer membrane structure for a long time. Rehydration is a complex process requiring multiple vortexing and heating steps to achieve complete reconstitution and solubilization of lipid nanovesicle powder^17^. By contrast, the proposed lyophilization methods in the CSF system using LPA can stabilize lipid bilayer membranes and prevent fusion, aggregation, and leakage. Furthermore, the process is comparatively simple; the lipid nanovesicle solution is frozen at − 75 °C in a liquid medium (isopropanol) during the freezing step without the need for any complex device or process.

## Conclusion

Lyophilization using CSF with LPA facilitates the powderization of lipid nanovesicles without agglomeration and retains the intrinsic physicochemical properties, including size distribution, structure, as well as membrane fluidity and polarity, after rehydration. We believe that the proposed method performs optimally in terms of processing and long-term storage of lipid nanovesicles, enhancing its applicability in the chemical, pharmaceutical, and food industries. Furthermore, this method can be applied to improve the original activity and durability of bioactive compounds with lipid nanocarriers.

## Supplementary Information

Supplementary Information.
