# Supplementary material for: Controlled rate slow freezing with lyoprotective agent to retain the integrity of lipid nanovesicles during lyophilization
Source: Sci Rep. 2021 Dec 21;11:24354. doi: 10.1038/s41598-021-03841-4 (PMC8692592; doi:10.1038/s41598-021-03841-4)
Supplement: Supplementary file 1 — Supplementary Information. [file 41598_2021_3841_MOESM1_ESM.docx]

**Supplementary material**

**Controlled rate slow freezing with lyoprotective agent to retain the integrity of lipid nanovesicles during lyophilization**

Eunhye Yang, Hyunjong Yu, SungHak Choi, Kyung-Min Park, Ho-Sup Jung^*^, and Pahn-Shick Chang^*^

**Corresponding author**

Ho-Sup Jung, jhs@snu.ac.kr; Pahn-Shick Chang, pschang@snu.ac.kr

**
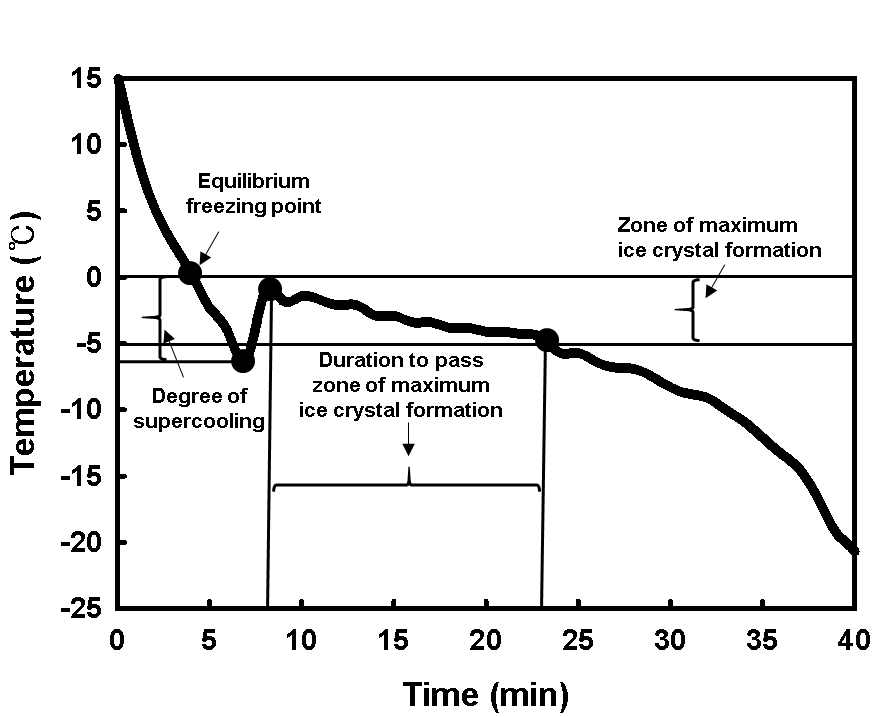
**

**Supplementary Fig. S1.** Freezing curve of a lipid nanovesicle solution obtained during conventional freezing, showing the supercooling and freezing rates.


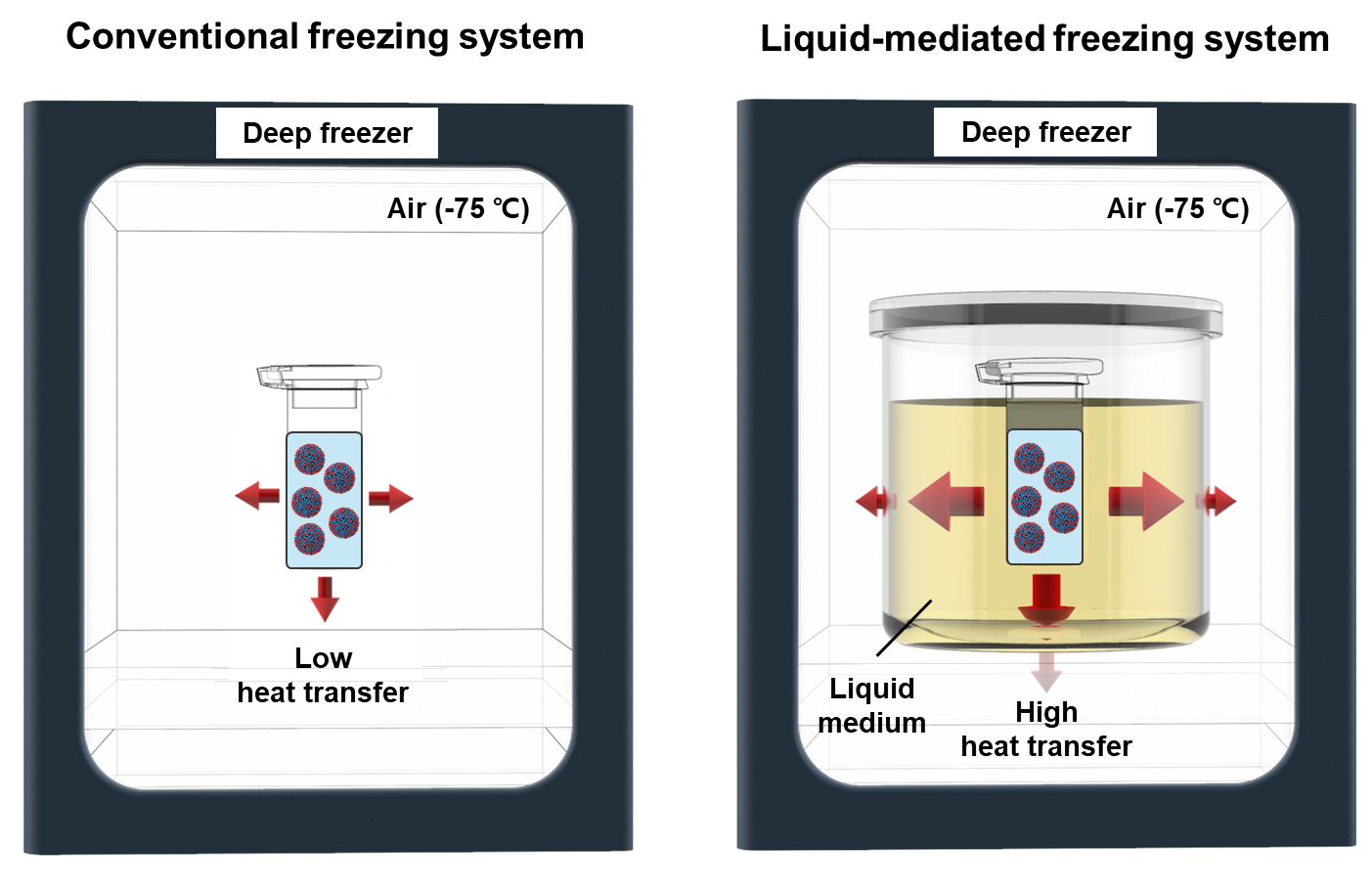
 **Supplementary Fig. S2.** Schematic illustration of a conventional freezing system and a liquid-mediated freezing system.

**
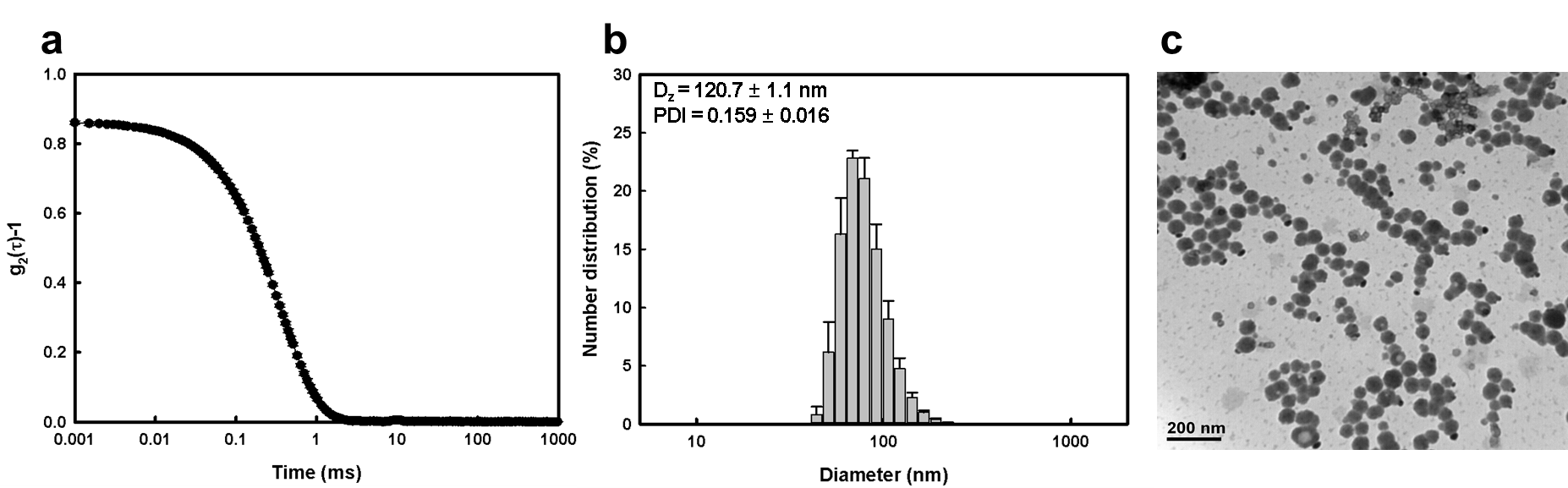
**

**Supplementary Fig. S3. (a)** Intensity correlation curve, and (b) size distribution (Z-average diameter and polydispersity index) and **(b)** transmission electron microscopy of intact lipid nanovesicles.

**
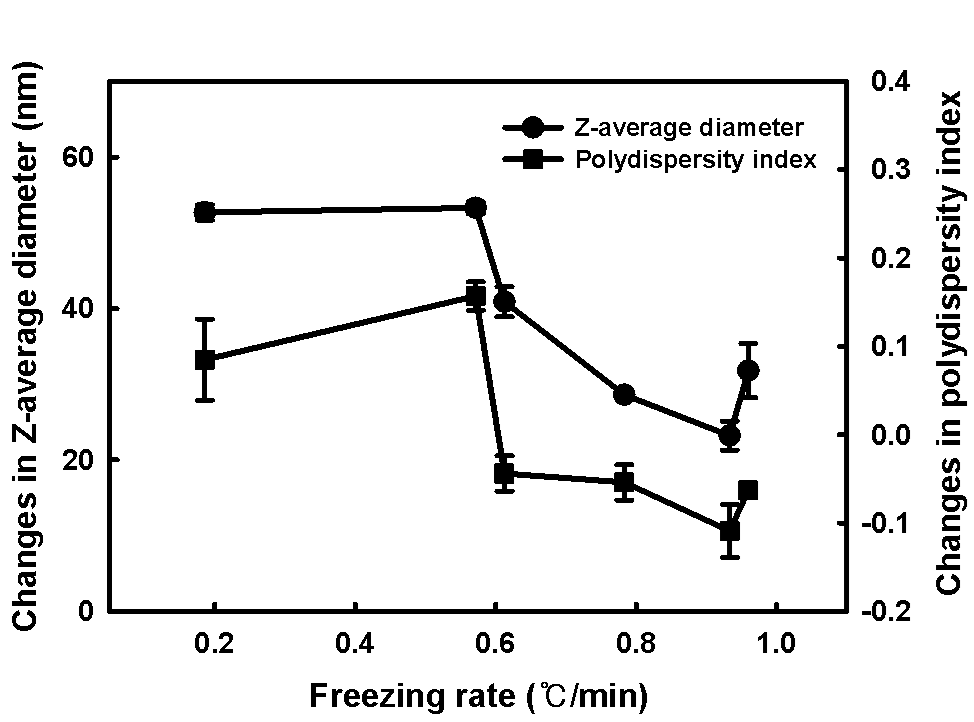
Supplementary Fig. S4.** Changes in size distribution (Z-average diameter and polydispersity index) of lyophilized lipid nanovesicles at different freezing rate and intact lipid nanovesicles prior to lyophilization.

**
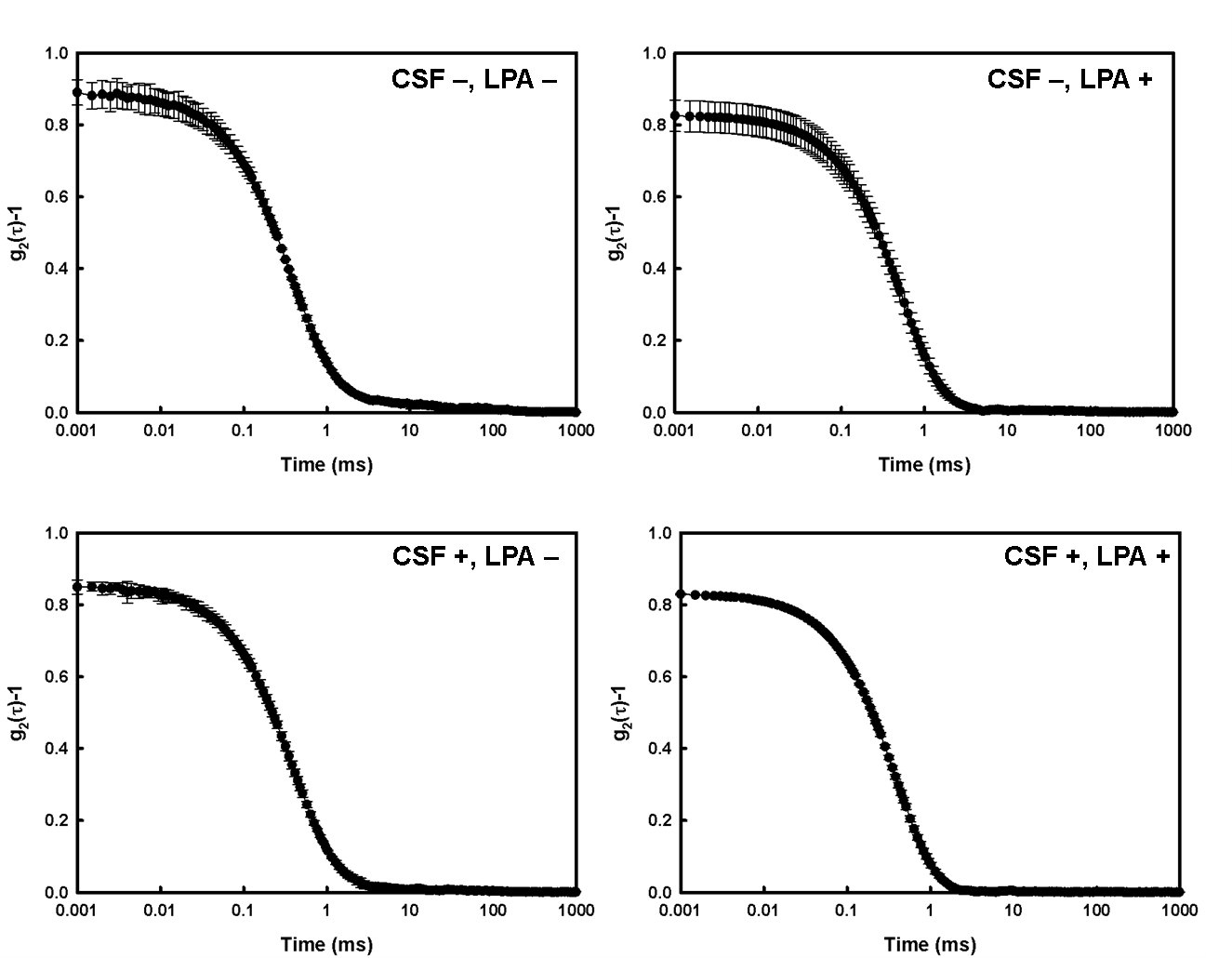
**

**Supplementary Fig. S5.** Intensity correlation curves of lipid nanovesicle powders after rehydration.

**Supplementary Table S1.** Specific heat capacity of various medium used in the LMF system at 0^o^C and freezing rate of the CF and LMF system using each medium.

| **Freezing system (medium)** | **Specific heat capacity  of medium (J/℃∙g)** | **Freezing rate (℃/min)** |
| --- | --- | --- |
| **Conventional freezing (air)** | **1.000** [**^1^**](#_ENREF_1) | **0.186 ± 0.076^a^** |
| **LMF (acetone)** | **2.114** [**^2^**](#_ENREF_2) | **0.571 ± 0.086^b^** |
| **LMF (*n*-propanol)** | **2.186** [**^3^**](#_ENREF_3) | **0.613 ± 0.058^b^** |
| **LMF (ethanol)** | **2.270** [**^4^**](#_ENREF_4) | **0.783 ± 0.024^c^** |
| **LMF (isopropanol)** | **2.475** [**^5^**](#_ENREF_5) | **0.933 ± 0.016^cd^** |
| **LMF (methanol)** | **2.567** [**^5^**](#_ENREF_5) | **0.966 ± 0.056^d^** |

Data are presented as means with standard deviations (*n*=3). Different letters indicate significant differences at *p* < 0.05 based on Tukey post-hoc analysis.

**References**

1 Arroyo, M., Sánchez-Montero, J. M. a. & Sinisterra, J. V. Thermal stabilization of immobilized lipase B from *Candida antarctica* on different supports: Effect of water activity on enzymatic activity in organic media. *Enzyme Microb. Technol.* **24**, 3-12, doi:10.1016/S0141-0229(98)00067-2 (1999).

2 Kelley, K. K. The heat capacities of isopropyl alcohol and acetone from 16 to 298 K. and the corresponding entropies and free energies. *J. Am. Chem. Soc.* **51**, 1145-1150, doi:10.1021/ja01379a022 (1929).

3 van Miltenburg, J. C., Berg, v. d. & K., G. J. Heat capacities and derived thermodynamic functions of 1-propanol between 10 K and 350 K and of 1-pentanol between 85 K and 370 K. *J. Chem. Eng. Data* **49**, 735-739, doi:10.1021/je0499768 (2004).

4 Miyazawa, T., Kondo, S., Suzuki, T. & Sato, H. Specific heat capacity at constant pressure of ethanol by flow calorimetry. *J. Chem. Eng. Data* **57**, 1700-1707, doi:10.1021/je2013473 (2012).

5 Katayama, T. Heats of mixing, liquid heat capacities and enthalpy-concentration charts for methanol-water and iso-propanol-water systems. *Chem. Eng.* **26**, 361-372, doi:10.1252/kakoronbunshu1953.26.361 (1962).
